# Docking study of novel antihyperlipidemic thieno[2,3-*d*]pyrimidine; LM-1554, with some molecular targets related to hyperlipidemia - an investigation into its mechanism of action

**DOI:** 10.1186/2193-1801-3-628

**Published:** 2014-10-24

**Authors:** Vijay M Khedkar, Nikhilesh Arya, Evans C Coutinho, Chamanlal J Shishoo, Kishor S Jain

**Affiliations:** Department of Pharmaceutical Chemistry, Bombay College of Pharmacy, Mumbai, 400 098 Maharashtra India; Department of Pharmaceutical Chemistry, Sinhgad Institute of Pharmaceutical Sciences, Lonavala, Pune, 410 401 Maharashtra India; Department of Chemistry, Banasthali University, Tonk, 304 022 Rajasthan India; B.V. Patel Pharmaceutical Education and Research Development (PERD) Centre, S.G. Highway, Thaltej, Ahmedabad, 380 054 Gujarat India

**Keywords:** Docking experiments, Antihyperlipidemic, 2-chloromethylthieno[2,3-*d*]pyrimidine, LM-1554, Molecular targets

## Abstract

An investigation into the mechanism of antihyperlipidemic action of 2-chloromethyl-5,6,7,8-tetrahydrobenzo(*b*)thieno[2,3-*d*]pyrimidin-4(3*H*)-one (LM-1554) was carried out through docking experiments with six different molecular targets; Niemann Pick C1 Like1 protein (NPC1L1), ATP citrate lyase (ACL), C-reactive protein (CRP), lanosterol 14*α*-demethylase (LDM), squalene synthase (SqS) and farnesiod X-receptor (FXR) known to be implicated in the physiology of hyperlipidemia. The interactions of LM-1554 were compared with the interactions of their respective co-crystallized native ligands at the active sites of these receptors. These comparisons are based on their docking parameters, as well as, types of interactions and vicinity with various amino acids in the active site pockets. The interaction of LM-1554 with the target, NPC1L1 has been found to be the quite favourable as compared to those with the other targets assessed in this study.

**Electronic supplementary material:**

The online version of this article (doi:10.1186/2193-1801-3-628) contains supplementary material, which is available to authorized users.

## Introduction

Atherosclerosis characterised by degenerative changes in the intima of medium and large arteries, is one of the main causes underlying cardiovascular disorders (CVD) and stroke which, are responsible for significant mortality, worldwide (McGill 1985; Overturf and Loose-Mitchell 1992; Ghatak and Asthana 1995; Schwandt 1990; Tiwari et al. 2006; Gordon et al. 1989; Go et al. 2014). Lowering of lipid levels in the blood is one of the major approaches to prevent atherosclerosis and thereby, CVD and stroke. Drugs currently used in therapy to treat hyperlipidemia, have several drawbacks. Medicinal chemists worldwide are routinely engaged in the discovery and development of newer molecules which can act differently and more effectively than the drugs presently employed in therapy. Thus, newer molecular targets related to hyperlipidemia are routinely exploited in the pursuit for discovering better, effective and safer drugs (Jain et al. 2010; Arya et al. 2014).

**Figure 1:**
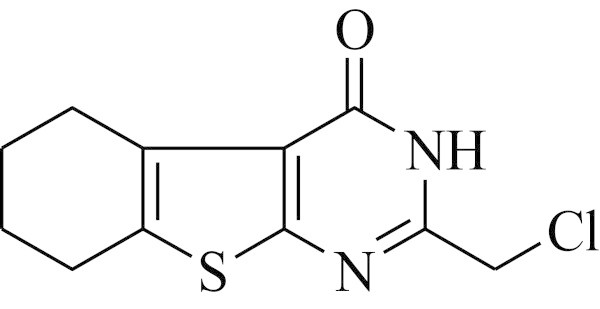
**LM-1554- The title compound.** Structure of LM-1554.

Thienopyrimidines have exhibited a variety of pharmacological activities. Thieno[2,3-*d*]pyrimidine 2-propionic acids (Shiroki 1976), 2-mercapto[2,3-*d*]pyrimidin-4-ones (Sauter 1972) and 2-substitutedmethylthieno[2,3-*d*]pyrimidine-4(3*H*)-ones (Shishoo et al. 1990; Jain et al. 2011) have been reported to exhibit good antihyperlipidemic activity. One of these compounds, 2-chloromethyl-5,6,7,8-tetrahydrobenzo(*b*)thieno[2,3-*d*]pyrimidin-4(3*H*)-one (LM-1554) (CAS # 89587-03-3) (Figure 1) was found to be promising, when evaluated in various animal models, employing different protocols of evaluation at different dose levels. Drugs used as reference standards in these studies were gemfibrozil, clofibrate, riboflavin tetrabutyrate, ezetimibe. Further, the acute and chronic toxicity studies of this compound indicated it to be considerably safe with high LD_50_ values (Shishoo et al. 1990, 1981, 1996; Jain et al. 2011; Arya 1985; Kathiravan et al. 2007).

This compound during its pharmacokinetic evaluation was found to be poorly absorbed through the gastrointestinal tract (Shishoo et al. 1997; Jain et al. 2007). Interestingly, it was found to be active orally, but inactive when given through parenteral route. This indicated its probable site of action to be at the surface of the GIT (something similar to the bile acid sequestering agents). QSAR studies undertaken on its analogs revealed the electronic parameter to be positively contributing to the antihyperlipidemic activity of these compounds. Electron withdrawing groups (EWG) attached at the 2-methyl substituent of these compounds, enhanced the activity (Shishoo et al. 1996; Kathiravan et al. 2007). All these interesting observations aroused our interest to probe into the pharmacodynamics of this compound.

Bioinformatics tools, like molecular docking experiments, which involve study and analysis of ligand-receptor interactions, play important role in identifying the molecular targets (receptors) for different ligands. We have periodically reviewed some such novel molecular targets for antihyperlipidaemic drug research (Jain et al. 2010; Arya et al. 2014). It was thought worthwhile, to assess the interaction of compound LM-1554, with few such molecular targets through, its *in silico* docking experiments and gain some insight on its probable mechanism of action. Six such molecular targets related to hyperlipidaemia were selected for this study. These were, Niemann Pick C1 like1 protein (NPC1L1), ATP citrate lyase (ACL), C-reactive protein (CRP), lanosterol 14α-demethylase (LDM), squalene synthase (SqS) and farnesiod X-receptor (FXR). The X-ray crystal structures of these targets complexed with their respective co-crystallized native ligands were available from the RSCB-Protein Data Bank (PDB).

NPC1L1 (PDB ID: 3QNT) (http://www.rcsb.org/pdb/explore/explore.do?structureId=3QNT), is an established molecular target for the cholesterol lowering drug ezetimibe. It plays an important role in the intestinal absorption of cholesterol. Inhibition or depletion of NPC1L1 reduces intestinal cholesterol absorption, resulting in reduction of plasma cholesterol levels (Kwon et al. 2011; Ge et al. 2008; Weingless et al. 2008; Calvo et al. 2005 and Kathiravan et al. 2009).

ACL (PDB ID: 3MWD) (http://www.rcsb.org/pdb/explore/explore.do?structureId=3MWD) is responsible for the supply of acetyl-CoA required for the biosynthesis of both cholesterol, as well as, fatty acids. Due to this its inhibition is considered to be more efficacious in correcting mixed hyperlipidemia as compared to that by the statins (Groot et al. 2003; Enache 2008; Chu et al. 2010 and Knowles et al. 1974).

CRP (PDB ID: 1B09) (http://www.rcsb.org/pdb/explore/explore.do?structureId=1B09) selectively binds with LDL, particularly the damaged LDL and gets deposited in the atherosclerotic plaques hastening the process of atherosclerosis. Further, aggregated and/or ligand-complexed CRP can be pro-inflammatory and is co-deposited with activated complements in all acute myocardial infarction lesions. Human CRP and its complements increase final myocardial infarction size in experimental models thus, making it a therapeutic target for decelerating the atherosclerotic plaque build-up process (Ridker 2003; Pepys et al. 2006; Libby et al. 2002 and Lowe 2005).

LDM (PDB ID: 3LD6) (http://www.rcsb.org/pdb/explore/explore.do?structureId=3LD6), a cytochrome P_450_ enzyme complex is responsible for catalysing an early step in cholesterol biosynthesis, namely the removal of the l4*α*-methyl group of lanosterol. Though, inhibitors of fungal LDM are used in therapy as antifungal agents, inhibitors of human LDM are not only of interest as mechanistic probes of the enzyme, but also as potential therapeutic agents for treatment of hypercholesterolemia (Strushkevich et al. 2010 and Gibbons 2002).

SqS (PDB ID: 1EZF) (http://www.rcsb.org/pdb/explore/explore.do?structureId=1EZF), catalyzes the biosynthesis of squalene, a key cholesterol precursor, through a reductive dimerization of two farnesyl diphosphate (FPP) molecules. Thus, SqS is an attractive target for therapeutic intervention of hyperlipidemia (Pandit et al. 2000; Nikitakis and Kourounakis 2011).

FXR (PDB ID: 1OSH) (http://www.rcsb.org/pdb/explore/explore.do?structureId=1OSH) functions as a bile acid (BA) sensor, coordinating cholesterol metabolism, lipid homeostasis and absorption of dietary fats as well as, vitamins. It plays an important role in maintaining bile acid and cholesterol homeostasis. In addition, activation of FXR lowers plasma triglyceride levels (Downes et al. 2003; Zhang et al. 2006 and Bailey et al. 2004). Due to these reasons, FXR becomes an attractive molecular target for indirect control of lipid levels.

## Results and discussion

**Figure 2:**
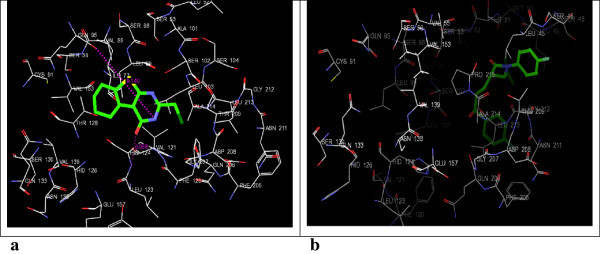
**3D-docking of LM-1554 with the molecular target NPC1L1. a**. 3D-docking of LM-1554 into PDB structure of NPC1L1. **b**. 3D-docking of ezetimibe into PDB structure of NPC1L1.

**Figure 3:**
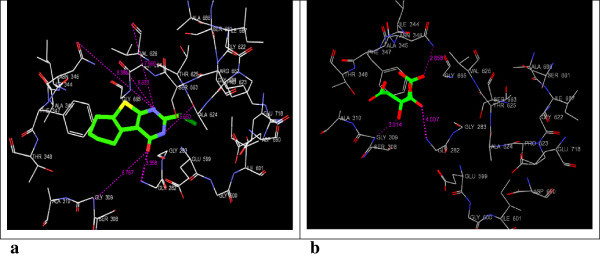
**3D-docking of LM-1554 with the molecular target ACL. a**. 3D-docking of LM-1554 into PDB structure of ACL. **b**. 3D-docking of native ligand, citric acid, into PDB structure of ACL.

**Figure 4:**
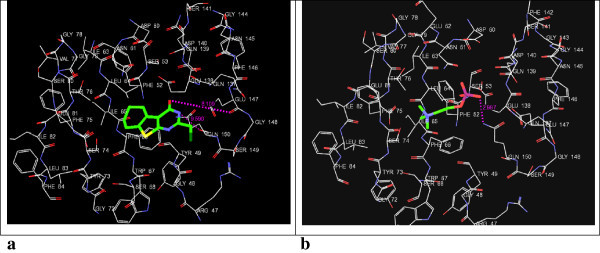
**3D-docking of LM-1554 with the molecular target CRP. a**. 3D-docking of LM-1554 into PDB structure of CRP. **b**. 3D-docking of native ligand, phosphocholine, into PDB structure of CRP.

**Figure 5:**
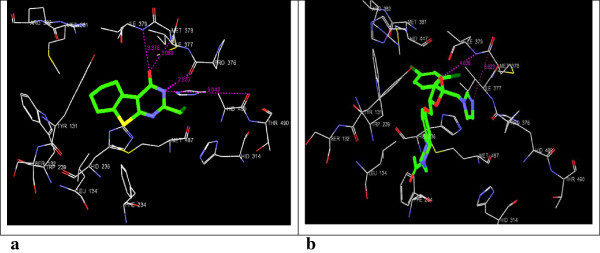
**3D-docking of LM-1554 with the molecular target LDM. a**. 3D-docking of LM-1554 into PDB structure of LDM. **b**. 3D-docking of native ligand, ketoconazole, into PDB structure of LDM.

**Figure 6:**
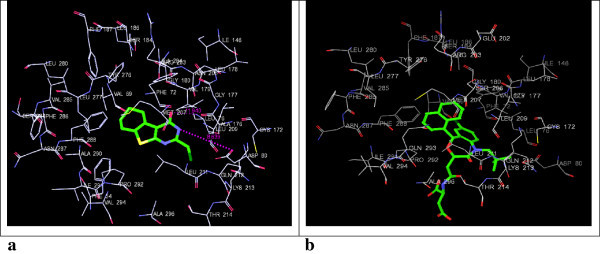
**3D-docking of LM-1554 with the molecular target SqS. a**. 3D-docking of LM-1554 into PDB structure of SqS. **b**. 3D-docking of native ligand,*N*-{2-[*trans*-7-chloro-1-(2,2-dimethylpropyl)-5-naphthalen-1-yl-2-oxo-1,2,3,5-tetrahydrobenzo[*e*][1,4]oxazepin-3-yl]acetyl}aspartic acid, into PDB structure of SqS.

**Figure 7:**
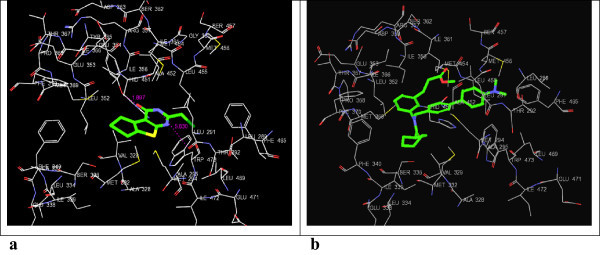
**3D-docking of LM-1554 with the molecular target FXR. a**. 3D-docking of LM-1554 into PDB structure of FXR. **b**. 3D-docking of native ligand, Fexaramine, into PDB structure of FXR.

By using Glide the docking simulations in the active sites of 3QNT, 3MWD, 1B09, 1EZF, 3LD6 and 1OSH were performed. LM-1554, as well as, their respective ligands (specified in Table 1), were docked in the active sites of these target protein structures and the best possible binding modes were obtained (Figures 2, 3, 4, 5, 6 and 7). Their corresponding docking scores, docking energy values, per residue interactions are listed in Table 1.

**Table 1:** **Data for the docking interactions of LM-1554 and respective ligands at the active sites of various molecular targets**

Sr. No.	Target protein (PDB-ID)	Ligand	Docking score	Energy (kcal/mol)			Interactions with aminoacid residues ^a^		
				Docking	Evdw	Ecoul	van der Waals (kcal/mol)	Electrostatic (kcal/mol)	H-bonding (kcal/mol) [Å]
1a.	**NPC1L1 (3QNT)**	LM-1554	-7.85	-33.52	-27.46	-6.06	ILE-218^b^ (-1.12)	ASN-127^b^ (-1.16)	HIS-124 (-0.87) [6.058]
							LEU-216^b^ (-1.60)	HIS-124 (-1.50)	GLN-95 (-0.01) [9.140]
							PRO-215^b^ (-2.50)	SER-102 (-3.08)	
							THR-128 (-1.77)	LEU-99 (-0.26)	
							ASN-127^b^ (-1.55)	SER-98 (-0.09)	
							HIS-124 (-3.24)		
							LEU-103 (-1.55)		
1b.	**NPC1L1 (3QNT)**	Ezetimibe^c^	-6.31	-32.35	-24.57	-7.77	ILE-218^b^ (-0.03)	ASN-127^b^ (-0.03)	No H-bonding observed.
							LEU-216^b^ (-0.09)	HIS-124 (0.04)	
							PRO-215 (-0.85)	SER-102 (1.30)	
							THR-128^b^ (-0.01)	LEU-99 (0.03)	
							ASN-127^b^ (-0.01)	SER-98 (-0.06)	
							HIS-124 (-0.05)		
							LEU-103 (-0.44)		
2a.	**ACL (3MWD)**	LM-1554	-6.39	-32.82	-28.86	-3.96	GLY-688 (-1.27)	GLY-665 (-2.02)	GLY-664 (-0.73) [5.63]
							GLY-665 (-3.41)	ALA-624 (-1.23)	VAL-626 (-0.62) [7.96]
							SER-663 (-3.75)	GLY-283 (-1.70)	ALA-624 (-0.14) [5.65]
							ARG-662^b^ (-1.48)		ASN-346 (-1.00) [8.39]
							PHE-347 (-3.52)		GLY-309 (-0.47) [6.77]
							ASN-346 (-2.46)		GLY-282 (-1.00) [3.36]
							ALA-345 (-1.84)		
							GLY-282 (-1.04)		
2b.	**ACL (3MWD)**	Citric acid^d^	-6.52	-30.43	-25.77	-4.65	GLY-688^b^ (-0.01)	GLY-665 (-2.02)	ASN-346 (-1.00) [2.858]
							GLY-665 (-1.44)	ALA-624 (0.69)	GLY-309 (-0.31) [3.014]
							SER-663 (-0.36)	GLY-282 (-2.26)	GLY-282 (-0.50) [4.007]
							ARG-662^b^ (-0.03)		
							PHE-347 (-3.29)		
							ASN-346 (-1.30)		
							ALA-345 (-2.92)		
							GLY-282 (-0.46)		
3a.	**CRP (1B09)**	LM-1554	-6.43	-17.49	-5.04	-12.45	GLN-150 (-1.16)	GLN-150 (-2.33)	GLN-150 (-1.00) [9.590]
							SER-149 (-0.03)	GLU-147 (-1.23)	GLU-147 (-0.50) [8.109]
							GLU-138 (-2.28)		
							GLU-81 (-3.41)		
							ASN-61 (-2.12)		
3b.	**CRP (1B09)**	Phosphocholine^d^	-6.83	-26.30	-8.612	-17.68	GLN-150 (-0.88)	GLN150 (-5.95)	GLN-150 (-0.50) [2.997]
							SER-149 (-0.03)	GLU147 (-0.93)	
							GLU-138 (-2.44)		
							GLU-81 (-2.20)		
							ASN-61 (-2.29)		
4a.	**LDM (3LD6)**	LM-1554	-6.73	-32.40	-26.30	-6.37	MET-487 (-4.86)	MET-487 (-0.98)	HIS-489 (-1.00) [9.047]
							MET-378 (-1.76)	MET-378 (-1.22)	ILE-379 (-0.19) [3.376]
							ILE-377 (-2.40)	PRO-376 (-0.15)	MET-378 (-1.00) [3.080]
							PRO-376 (-1.23)		PRO-376 (-1.00) [2.830]
							HIS-314 (-1.81)		
							TRP-239 (-1.13)		
							PHE-234 (-1.41)		
							LEU-134 (-1.61)		
							TYR-131 (-1.69)		
4b	**LDM (3LD6)**	Ketoconazole^d^	-8.87	- 55.26	-52.75	-2.51	MET-487 (-3.71)	MET-487 (0.59)	ILE 379 (-0.60) [4.636]
							MET-378 (-2.39)	MET-378 (-2.23)	MET 378 (-1.00) [5.623]
							ILE-377 (-2.39)	PRO-376 (-0.39)	
							PRO-376 (-1.16)		
							HIS-314 (-0.39)		
							TRP-239 (-3.62)		
							PHE-234 (-1.69)		
							LEU-134 (-0.97)		
							TYR-131 (-3.50)		
5a.	**SqS (1EZF)**	LM-1554	-7.18	-31.29	-28.30	-2.99	PRO-292 (-1.95)	ALA-176 (-0.58)	ALA-176 (-0.97) [10.940]
							PHE-288 (-3.05)	ASP-80 (-1.22)	ASP-80 (-0.50) [8.630]
							LEU-211 (-2.67)		
							GLY-208^b^ (-1.49)		
							MET-207 (-1.92)		
							LEU-183^b^ (-2.91)		
							GLY-180 (-1.29)		
							VAL-179 (-2.29)		
							ALA-176 (-0.58)		
							PHE-54 (-1.69)		
5b.	**SqS (1EZF)**	*N*-{2-[*trans*-7-chloro -1-(2,2-dimethylpropyl) - 5-naphthalen-1-yl-2-oxo-1,2,3,5-tetrahydrobenzo[e] [1,4]oxazepin-3-yl]-acetyl}aspartic acid^d^	-11.17	-67.24	-49.80	-17.44	PRO-292 (-2.23)	ALA-176 ^b^ (-0.11)	No H-bonding observed
							PHE-288 (-3.81)	ASP-80 (-1.91)	
							LEU-211 (-3.56)		
							GLY-208^b^ (-1.39)		
							MET-207 (-2.82)		
							LEU-183^b^ (-3.37)		
							GLY-180 (-1.27)		
							VAL-179 (-4.20)		
							ALA-176^b^ (-1.08)		
							PHE-54^b^ (-4.53)		
6a.	**FXR (1OSH)**	LM-1554	-6.55	-29.62	-26.94	-2.68	TRP-473 (-1.72)	TYR-365 (-1.95)	TYR-365 (-1.00) [1.897]
							MET-454 (-1.37)		LEU-291 (-0.84) [5.830]
							HIS-451 (-1.44)		
							MET-369 (-1.77)		
							ILE-361 (-1.99)		
							ILE-356 (-1.18)		
							MET-332 (-1.52)		
							ALA-295 (-1.52)		
							MET-294 (-2.25)		
							THR-292 (-1.13)		
							LEU-291 (-5.20)		
6b.	**FXR (1OSH)**	Fexaramine^d^	-10.69	-43.71	-38.57	-5.14	TRP-473 (-0.94)	TYR-365^b^ (-0.17)	No H-bonding observed
							MET-454 (-1.92)		
							HIS-451 (-1.10)		
							MET-369 (-2.30)		
							ILE-361 (-2.58)		
							ILE-356 (-3.30)		
							MET-332 (-1.35)		
							ALA-295 (-0.74)		
							MET-294 (-4.17)		
							THR-292 (-0.82)		
							LEU-291 (-4.32)		

^a^All amino acid residues were within 5 Å from the ligand surface and 10 Å from the centroid of the ligands.

^b^These amino acid residues though not visible in the figures, were actually on the rear side of this 3D pose and were observed in the interaction energy tables,as well as, in other poses.

^c^Ezetimibe was taken as reference native ligand for better comparison.

^d^Native ligand as co-crystallized in the PDB 3D structures.

### Docking of LM-1554 into PDB structure of NPC1L1 (PDB ID: 3QNT)

The results obtained from the docking study indicated no significant difference in the binding modes of both the molecules, LM-1554 and ezetimibe in the same binding pocket in NPC1L1, defined by the amino acid residues, LEU-52, LEU-99, ALA-101, SER-102, HIS-124, THR-128, GLN-206, LEU-103, LEU-213, GLN-95, SER-98, and THR-209 (Figure 2a and b). The per residue interaction profile indicated the van der Waals contacts (-27.46 kcal/mol) to be more prevalent over the electrostatic contributions (-6.06 kcal/mol) in the overall binding of LM-1554 to NPC1L1. The compound made strongly favorable van der Waals interactions with ILE-218, LEU-216, PRO-215, THR-128, ASN-127, HIS-124 and LEU-103 residues in the active site. In addition to these interactions, LM-1554 was also found to be involved in favorable electrostatic interactions with ASN-127, HIS-124, SER-102, LEU-99 and SER-98, residues. The compound formed hydrogen bond interactions with the HIS-124 and GLN-95 residues of the active site. The ligand ezetimibe was also seen to be involved in favorable van der Waals as well as electrostatic interactions, with same residues, though the energies of interactions differed slightly, as compared with those of LM-1554 (Table 1, Sr. No. 1a & 1b). From the analysis of these docking interactions, the docking score of the LM-1554 (-7.85) was found to be better than that of the docking score for ezetimibe (-6.31). Also, the binding energy of LM-1554 (docking energy of -33.52 kcal/mol), at the active site of NPC1L1, compared well with the binding energy of ezetimibe at NPC1L1. All these observations indicated that LM-1554 probably exerted its antihyperlipidemic action through a mechanism similar to ezetimibe, *i.e.,* by inhibiting NPC1L1.

### Docking of LM-1554 into PDB structure of ATP-citrate lyase (3MWD)

LM-1554 when docked into the PDB structure of ACL (3MWD) occupied the pocket, defined by the amino acid residues GLY-688, GLY-665, SER-663, ARG-662, PHE-347, ASN-346, ALA-345, GLY-282, GLY-283, ALA-624, VAL-626, and GLY-309 (Figure 3a and b; Table 1, Sr. No. 2a & 2b). The per-residue interaction analysis showed LM-1554, involved in electrostatic interactions with the amino acid residues, GLY-665, ALA-624, and GLY-283 in the binding pocket of the enzyme. In addition, LM-1554 also formed hydrogen bonds with GLY-664 (5.633 Å), VAL-626 (7.960 Å), ALA-624 (5.650 Å), ASN-346 (8.386 Å), GLY-309 (6.767 Å) and GLY-282 (3.358 Å). The van der Waals interactions of LM-1554 were observed with some of the key amino acid residues; GLY-688, GLY-665, SER-663, ARG-662, PHE-347, ASN-346, ALA-345 and GLY-282. The docking score for LM-1554 was found to be -6.39, as against -6.52 observed for the native ligand. The docking energy of interaction for LM-1554 at the active site was -32.82 kcal/mol (van der Waals = -28.86 kcal/mol and Coulombic = -3.96 kcal/mol). The comparable docking scores and energies, as well as good extent of H-bonding indicated, ACL also to be a likely target for LM-1554.

### Docking of LM-1554 into the PDB structure of C-reactive protein (CRP)

LM-1554 when docked on this molecular target was found to be anchored in the active pocket through two hydrogen bonds with GLN-150 and GLU-147, at distances of 9.590 Å and 8.109 Å, respectively (Figure 4a and b; Table 1, Sr. No. 3a & 3b). The compound also formed electrostatic interactions with the GLN-150 and GLU-147 residues as well as, van der Waals contacts with GLN-150, SER-149, GLU-138, GLU-81 and ASN-61 residues. However, the comparison of its docking score (-6.43) and docking energy (-17.49 kcal/mol) values with those for the native ligand having docking score (-6.83) and docking energy (-26.30 kcal/mol) values, suggested CRP to be the less likely molecular target for LM-1554.

### Docking of LM-1554 into PDB structure of LDM (PDB ID: 3LD6)

Docking interactions of the compound, LM-1554, into the active site of human LDM (CYP51) was analysed. The compound was involved in hydrogen bond interactions with the key amino acids of the active site; HIS-489, ILE-379, MET-378 and PRO-376. The interaction distances were 9.047 Å, 3.376 Å, 3.080 Å and 2.830 Å, respectively (Figure 5a and b; Table 1, Sr. No. 4a & 4b). Besides significant van der Waals interactions with the residues MET-487, MET-378, ILE-377, PRO-376, HIS-314, TRP-239, PHE-234, LEU-134 and TYR-131; LM-1554 also showed some electrostatic interactions with MET-487, MET-378, PRO-376 residues of the enzyme active site. However, both the docking score (-8.87) as well as docking energy (-55.26 kcal/mol) for the interaction of the native ligand at the active site were seen to be more favourable as compared to that of LM-1554 having docking score (-6.73); docking energy (-32.40 kcal/mol), indicating, the later to be a less likely ligand for human LDM.

### Docking of LM-1554 into PDB structure of SqS (PDB ID: 1EZF)

Docking of LM-1554 into PDB structure of SqS revealed it to be interacting through hydrogen bonds as well as electrostatic interactions with ALA-176 and ASP-80 residues of the active site (Figure 6a and b, Table 1, Sr. No. 5a & 5b). The van der Waals interactions with key amino acid residues, PRO-292, PHE-288, LEU-211, GLY-208, MET-207, LEU-183, GLY-180, VAL-179, ALA-176, PHE-54, in the binding site of SqS were observed for both ligands. However, both, the docking score (-11.17), as well as, docking energy (-67.24 kcal/mol) for the interactions for the native ligand with SqS were seen to be more favourable as compared to that of LM-1554 having docking score (-7.18); docking energy (-31.29 kcal/mol), during its interactions with SqS, indicating, LM-1554 not a likely ligand for SqS.

### Docking of LM-1554 into PDB structure of FXR (PDB ID: 1OSH)

LM-1554 as well as the respective native ligand for FXR, were docked into the active pockets of the FXR for the comparative assessment of the favourability of their interactions at the active site (Figure 7a and b, Table 1, Sr. No. 6a & 6b). LM-1554 besides exhibiting van der Waals contacts with TRP-473, MET-454, HIS-451, MET-369, ILE-361, ILE-356, MET-332, ALA-295, MET-294, THR-292 and LEU-291 residues in the active pocket, also exhibited electrostatic interactions with the TYR 365 residue and hydrogen bonding with TYR-365 (1.897 Å), as well as, LEU-291 (5.830 Å). However, the native ligand showed comparatively better interactions with the above mentioned residues as reflected from its comparatively favorable docking score (-10.69) and energy values (-43.71 kcal/mol), indicating, LM-1554 having docking score (-6.55); docking energy (-29.62 kcal/mol), less likely to be a ligand for FXR.

A perusal of the docking scores of LM-1554 at all the above six molecular targets as compared to their respective ligands (Table 1), revealed its better docking interactions at NPC1L1. Further, on the basis of comparative docking energies, ACL also appeared to be its other favorable target.

## Conclusions

LM-1554 (2-chloromethyl-5,6,7,8-tetrahydrobenzo(*b*)thieno[2,3-*d*]pyrimidin-4(3*H*)-one; CAS #89587-03-3), which had shown promising antihyperlipidemic activity in its preclinical evaluation and also found to be safe in its toxicity studies warranted an investigation into its pharmacodynamics. The technique of molecular docking was utilised for analysing the orientation of conformations and poses, as well as, assessing favourability of interactions of LM-1554 into the binding pockets of six different molecular targets related to hyperlipidemia. This was done to gain some insights in its probable mechanism of action, as an antihyperlipidemic entity. Concluding from the results (Figures 2, 3, 4, 5, 6 and 7, Table 1), the compound seemed to be acting through the inhibition of NPC1L1. ACL also could be its molecular target to some extent. Thus, on this basis, selective *in vitro* assays involving these two targets could now be the next step to confirm its mechanism of action. LM-1554, at dose level of 10 mg/kg. *p.o.* was found to exhibit good antihyperlipidemic activity, as seen by two different evaluation protocols.

## Computational details

All the molecular docking analyses for LM-1554 and the native ligands with the molecular targets were performed using the Glide® molecular modeling package (Schrödinger, Inc., USA) running on an Intel Xeon based system with the Linux Enterprise OS.

The starting coordinates of the protein structures - Niemann Pick C1 like1 (NPC1L1) (PDB ID: 3QNT), ATP-citrate lyase (ACL) (PDB ID: 3MWD), C-reactive protein (CRP) (PDB ID: 1B09), squalene synthase (SqS) (PDB ID: 1EZF), human lanosterol 14α-demethylase (CYP51) (3LD6) and farnesoid X receptor (FXR) (PDB ID: 1OSH) were obtained from the RCSB Protein Data Bank and further modified for the docking calculations. The protein structures were prepared by running the protein preparation wizard and applying the force field, OPLS-2005. Thereafter, removal of crystallographic water molecules and addition of hydrogens to the structures corresponding to pH 7.0 was done. The most likely positions of hydroxyl and thiol hydrogen atoms, protonation states and tautomers, as well as, the Chi ‘flip’ assignments for the amino acid residues were selected using the protein assignment script. After assigning appropriate charge and protonation states, the prepared structures were further refined by subjecting to energy minimization until the average root mean square deviation (r.m.s.d.) reached 0.3 Å.

For precision and accuracy of the docking protocols, the co-crystallized ligands were extracted from the crystal structures of ACL, SqS, FXR, CRP and LDM and were re-docked using Glide docking algorithm in its XP mode. A good agreement was observed between the localization of the native ligands upon docking and as such in the co-crystallized structures (r.m.s.d. <1.0 Å).

In case of NPC1L1, eventhough, the its crystal structure with ligand *N*-acetylglucosamine complexed with it was available (PDB ID: 3QNT) (Kwon et al. 2011), we considered ezetimibe, a known cholesterol lowering drug acting on this target as a reference ligand in the present study for a better comparison (Arya et al. 2013).

The initial 3D structure LM-1554 and ezetimibe were built using the Maestro module (Schrödinger, Inc., USA) and optimized by the Lig-Prep module (Schrödinger Suite). The partial charges were assigned using the OPLS2005 (Optimized Potentials for Liquid Simulations) force-field with target pH of 7.0. The ligand geometries were refined through energy minimization (LBFGS method) to a target gradient of 0.001 kcal/mol/Å.

With the protein and ligand in the correct form, the next step was the generation of the receptor-grid for defining the active pocket for docking using Glide (Schrödinger). All amino acids within 10 Ǻ of the co-crystallized ligand were included in the grid file generation. Default values were retained for the van der Waals scaling and partial charges were assigned from the input structure, rather than from the force field, by selecting the use input partial charges option.

Following the grid generation, LM-1554 and the respective native ligands were docked into all the aforementioned targets in separate docking experiments. The extra-precision (XP) scoring function in Glide was used to rank the docking poses and to evaluate the binding affinity of the LM-1554 as well as the native ligands for the respective targets. To analyze the mode of binding, the docked conformation with best Glide (XP) score was selected.

(More details on the docking protocol are provided in the Additional file 1).

## Electronic supplementary material

Additional file 1: **Supplementary data for docking protocol.** (DOC 64 KB)
