# Supplementary material for: Docking study of novel antihyperlipidemic thieno[2,3-d]pyrimidine; LM-1554, with some molecular targets related to hyperlipidemia - an investigation into its mechanism of action
Source: Springerplus. 2014 Oct 24;3:628. doi: 10.1186/2193-1801-3-628 (PMC4221561; doi:10.1186/2193-1801-3-628)
Supplement: Supplementary file 1 — Additional file 1: Supplementary data for docking protocol. (DOC 64 KB) [file 40064_2014_1336_MOESM1_ESM.doc]

Additional file 1 **Supplementary data for docking protocol**

Favourable positions, orientations and conformations of the ligand in the enzyme-binding pocket were searched *via* a series of hierarchical filters by the Glide algorithm. The shape and properties of the active site were represented on a grid by different sets of fields to provide progressively more accurate scoring of the ligand poses. The binding site was defined by a rectangular box (grid) confining the translations of the mass centre of the ligand. Thereafter, a set of initial ligand conformations was produced through an exhaustive search of the torsional minima, and the conformers were clustered in a combinatorial fashion. Each cluster was characterized by a common conformation of the ″core″ and an exhaustive set of ″rotamer group″ conformations, was docked as a single object in the first stage. The search began with a rough positioning and scoring phase that significantly narrowed the search space and reduced the number of poses to be further considered to a few hundred so that computationally expensive energy and gradient evaluations could be performed. Thereafter, the selected poses were minimized in the field of the receptor using OPLS-AA force field in conjunction with a distance-dependent dielectric model. The minimized poses generated by docking were scored using the Glide Extra-Precision (XP) scoring function equipped with a variety of force field-based parameters accounting for solvation and repulsive interactions, lipophilic, hydrogen bonding interactions, metal–ligand interactions, as well as, contributions from coulombic and van der Waals interaction energies, all incorporated in the empirical energy functions.
